# A global clustering of terrestrial food production systems

**DOI:** 10.1371/journal.pone.0296846

**Published:** 2024-02-14

**Authors:** Martin Jung, Timothy M. Boucher, Stephen A. Wood, Christian Folberth, Michael Wironen, Philip Thornton, Deborah Bossio, Michael Obersteiner

**Affiliations:** 1 International Institute for Applied Systems Analysis (IIASA), Laxenburg, Austria; 2 The Nature Conservancy, Arlington, Virginia, United States of America; 3 Yale School of the Environment, New Haven, United States of America; 4 Clim-Eat, c/o Netherlands Food Partnership, Utrecht, The Netherlands; 5 Environmental Change Institute, University of Oxford, Oxford, United Kingdom; Sichuan University, CHINA

## Abstract

Food production is at the heart of global sustainability challenges, with unsustainable practices being a major driver of biodiversity loss, emissions and land degradation. The concept of foodscapes, defined as the characteristics of food production along biophysical and socio-economic gradients, could be a way addressing those challenges. By identifying homologues foodscapes classes possible interventions and leverage points for more sustainable agriculture could be identified. Here we provide a globally consistent approximation of the world’s foodscape classes. We integrate global data on biophysical and socio-economic factors to identify a minimum set of emergent clusters and evaluate their characteristics, vulnerabilities and risks with regards to global change factors. Overall, we find food production globally to be highly concentrated in a few areas. Worryingly, we find particularly intensively cultivated or irrigated foodscape classes to be under considerable climatic and degradation risks. Our work can serve as baseline for global-scale zoning and gap analyses, while also revealing homologous areas for possible agricultural interventions.

## Introduction

The terrestrial surface of the Earth has a long history of human use across most geographic regions [1]. Today, global agricultural and livestock production accounts for the largest share of harvested biomass [2] and anthropogenic land use [3, 4], while being a major driver behind the destabilization of planetary boundaries [5]. In order not to jeopardize these planetary boundaries or new post-2020 policy targets (e.g., the SDGs, UNFCC or CBD), it becomes important to navigate the complex tradeoffs between agricultural production and safe-operating spaces for human health, biodiversity and the wider nexus [6–8]. Not all forms of agricultural production however are sustainable or productive everywhere, particularly in the context of nutrient management [9], conservation agriculture [10] or the ‘land sharing vs land sparing’ debate [11, 12]. To align possible interventions for improved land practices globally, we need to better understand where commonalities and differences between food production systems exist. Globally, food production is incredibly diverse. It can be spatially concentrated [4], driven by an often long history of use and socio-economic dynamics [1, 13], and varying in terms of its environmental impacts and sustainability [14]. Nevertheless, there are some regional commonalities with much of the world’s food being produced among biophysical and socioeconomic gradients and constraints [15]. Some of the most well-known examples include intensively irrigated ‘bread basket’ regions such as the Indus valley, the black Earth or *Chernozem* regions in the Russian Federation or the US Corn belt, which house a large part of the world’s contemporary food production [16]. Similarly, food production in other regions is characterized by dispersed small-scale farming, dominated by animal husbandry, or tree plantations [17]. Despite this heterogeneity, there likely exist homologous conditions of food production systems even across large distances and their identification could benefit the design of possible broad-scale interventions for sustainable agricultural intensification [18] and supporting knowledge and financing transfers towards transformative change in the food sector. Here, the concept of a ‘foodscape’ as distinct combinations of biophysical, production and socio-economic factors related to food production may be useful in identifying such homologous conditions.

The quest to identify commonalities of terrestrial land surface is not new and is usually undertaken through the concept of archetyping. Here different frameworks have created classifications of land systems as defined by patterns of land cover and land use at global [19–21] or regional scale [22–24]. Such classifications can be used to better understand similarities and differences in current trajectories of change [25, 26] or patterns of vulnerabilities [27]. Most of such classifications, however, are created for a particular need and are—owing to differences in spatial, temporal and thematic resolution—not necessarily adaptable for different use cases. For example, previous archetyping studies that aimed at mapping global land systems had the aim to primarily lay out the diversity of land systems in terms of land cover and land use [20, 21]. To our knowledge no such global archetypes exist that explicitly focus on different forms and intensities of global food production.

Archetyping food production systems would allow us to better understand and compare different and analogous types of food production globally. Knowing which factors broadly determine food production across regions can open up opportunities for bundling interventions and innovations in the food sector [28, 29]. There are many reasons behind yield gabs globally, with especially inefficient management practices are considered to be common [30], and addressing them can provide opportunities to reduce impacts and increase the resilience of food production. For example, it has been found that a higher diversity of crops and the inclusion of livestock in mixed systems can stabilize yields, improve nutritional value and biodiversity benefits, and increase resilience to extreme climatic events [31–34]. Similarly, there might be opportunities in increasing soil organic carbon stocks through altered crop rotations and tillage practices [35], which has been shown to increase crop yields and reduce yield variability to extreme weather events [36, 37]. Obviously, any such archetyping at the global scale is necessarily an approximation and can only reflect the broad similarities of food production at coarse grain. Yet, it can serve as an important first step in coming up with a scope of possible interventions and identification of homologous systems.

Advances in remote sensing techniques and statistical modelling have created a wealth of new global spatial information on the biophysical and socio-economic conditions of food production. Increasingly, high-resolution data on the distribution of land cover and soil types [38, 39] and land use are becoming available, the latter being able to identify factors related to farming such as crop production, field size, forest management or livestock density [16, 40–43]. Harmonized data sources such as the new Global Agro-Ecological Zones data (GAEZ v4, [15] being an integrated view of the potential suitability of agricultural production, yet critically miss aspects of land use and management. Despite the many uncertainties and thematic differences in global land-use data [44], we believe there is an opportunity to integrate these novel data sources to map broad classes that approximate foodscapes from a biophysical and socio-economic point of view.

In this work we collated and harmonized currently best available global spatial datasets on the biophysical and management properties of terrestrial food production systems (S1 Fig in S1 File). We then—in an unsupervised classification approach—used those datasets to identify global clusters of units with similar properties of food production, coined ‘foodscapes’. Our aim is to (a) identify broad classes of foodscapes that are comparable and consistent across biogeographic regions, (b) investigate the characteristics and heterogeneity of foodscape classes across scales and production factors, and (c) assess the various contemporary risk factors that these foodscape classes are exposed to. We note that this form of predominantly data-driven clustering highlights regions of similar distinctive characteristics, rather than areas described based on a priori defined legend or classification system. Rather than a “one-size-fits-all” approach, we envisage these clusters to be particularly useful in identifying homologue regions of global food production as well as for screening opportunities of possible interventions. All output layers are made openly available as part of this publication.

## Methods

### Experimental design and preparation of variables

Characteristics of global food production systems are widely determined by a series of geophysical and management related factors. Together with global and regional experts from the wider team at The Nature Conservancy we identified a minimal set of 59 global variables that are key characteristics of global food production systems (S1 Table in S1 File, see also below). This minimal set was drawn from an initial, broader list of known factors to influence food production, many of which included variables such as governance, value chains or informal norms, which however were not sufficiently quantified on a global scale. We chose the minimal set of variables included here based on data availability, data quality, interpretability and reduction of collinearities after visual examination (see statistical analyses). We separated all variables into two levels, one including biophysical and land-use variables and the other including management related variables. All data preparation was done at a target resolution of 5km loosely resembling the extent of a broader landscape in each area and the finest grain possible with global data (see also caveat section in discussion). As a template for further rasterization and downscaling we created a global land area and coastal cell mask based on the GADM dataset (https://gadm.org/), which we first rasterized at 1km resolution and then used to calculate the fraction of land area in any 5km grid cell. Variables were scaled (subtracted by their mean and divided by 1 standard deviation) for the analysis to ensure comparable units, and included either in binary or continuous form.

For terrestrial biophysical variables at level 1 we broadly considered data on topography, land cover and growing season (S1 Fig in S1 File). For topography we used data on the distribution of global landforms at 250m resolution [45]. Landform types were merged into either plains, hills or mountains and then fractionally aggregated to a 5km grid cell. To include information on soil type we considered farming related soil groups from the United States Department of Agriculture (USDA) soil database [39, 46], specifically Alfisols, Andisols, Entisols, Histosols, Inceptisols, Mollisols, Oxisols, Spodosols, Ultisols and Vertisols. For each of those soil groups we identified the dominant soil type through a majority aggregation from the original 250m to 5km grid cell. For land cover we used data from the Copernicus global land cover (CGLC, [38]) dataset for the year 2015. Here we used the predicted fractional cover of trees, shrubs, grasses, bare, crops and built-up covered land, which we aggregated (arithmetic mean) to the target resolution of 5km. We included data on the distribution of forest-related agroforestry [40], which although it is a management related variable, was primarily used to help separating mixed forests and cropland systems (but see [40]. Lastly, we considered data that represent the biophysical limits of vegetation growth, specifically the aridity index describing a continuum of humidity to aridity globally [47] and the global length of growing period in days per year from the Global Agro-Ecological Zoning dataset [48]. Data on aridity was aggregated to 5km and length of growing period data was resampled to 5km (original resolution 10km), while joining areas with a 365-day growing period into a single class. We also considered data on global climatic zones from the Köppen-Geiger climate system [49]; however, because of the large number of discrete classes those were not directly included in the analysis, but only considered for post-hoc identification and separation of dominant climatic zones (see overlay analysis).

For variables at level 2 for terrestrial land management we considered a series of global datasets related to management intensity or cropping practices. A key data source for crop groups was the latest global data from the Spatial Production Allocation Model (SPAM) for the year 2010 [16]. From the SPAM database we collated data on the physically and production area of all reported crop types across cropping technologies. We considered maize, rice, wheat, potato, cassava, soybean, sweet potato, yams, sorghum and plantains as “staple crops”, having some of the largest production globally. The total sum of staple crop production was finally resampled (bilinear) to a target resolution of a 5km grid cell. We combined these production estimates to identify hotspots of global food production, recognizing that especially oil crops are also used for feed and fuel [50]. In addition, we grouped all crops covered by SPAM into six crop groups: Cereals and Oil Crops, Tubers, Legumes and Pulses, Perennials, Vegetables, and Other crops (S2 Table in S1 File). These groupings were chosen based on similar cultivation forms, practices, or their commonly associated cultivation such as cereals and oil crops in animal feed production. We then calculated for each crop group and grid cell the total production area relative to the total area of cropland, resulting in a series of maps highlighting which crop group is predominantly grown in an area. Using data of physical area for all crops from SPAM we furthermore calculated the proportion of cropland for the year 2010 that is either irrigated or rainfed in a given grid cell, and furthermore estimated the diversity of crops in a given area by calculating a Shannon diversity index ($H\prime =-\sum_{i=1}^{crops}p_{i}\;ln(p_{i})$, where *i* is a given crop and *p*_*i*_ the physical area estimates). The resulting maps were then resampled (bilinear or nearest neighbor in case of binary data) to the target resolution of 5km.

We then linked the SPAM physical area estimates with data on tillage practices [43]. Based on expert feedback and similarities in effects on soil properties we grouped the six categories of predicted tillage into three groups: Conventional and Rotational tillage, Conservation and reduced tillage, and Traditional annual and rotational tillage practices. The resulting layer of dominant tillage practices was then resampled (nearest neighbor) to the target resolution of 5km. To separate areas that are single cropped we included the fraction of crops grown under a single cropping season only [51]. This data was created by aggregating (arithmetic mean) both irrigated and rainfed fractions of crops and then resampled (bilinear) to the target resolution of 5km.

Croplands with smaller fields often have particular cropping systems [52] and are predominantly cultivated by smallholders, although farm size and other landscape-related factors can also play a role. We used data on global field size distribution [53] to distinguish between very small fields (less than 0.64ha) to large fields (over 100ha). Instead of using the six binary classes on field size distribution in Lesiv et al. (2019), we converted them to a continuum per grid cell, using the bounded field size information in Lesiv et al. and the amount of area containing cropland according to Copernicus as additional information to assign in a heuristic manner the maximum average field size bounded by the available cropland area in a given grid cell.

In the most intensively used cropland high rates of nutrient fertilizers are commonly applied. To assess how strongly an area is on average fertilized we calculated the area-weighted nitrogen application rate across major crops in a given pixel using data on crop-specific nitrogen input from mineral fertilizer, manure, and atmospheric deposition [54]. For each crop we first calculated the fertilizer application volume and then summed up the resulting estimates across crops. Total nitrogen inputs were then weighted by total crop harvest area. Because of a few large outliers in some grid cells, caused by high manure application estimates, we applied a “winsorization” on the average estimate, capping the maximum possible nutrient application rate to the 95% percentile. The final map was then resampled (bilinear) to the target grid cell resolution of 5km.

Data on animal husbandry and grazing are notoriously difficult to obtain at high spatial resolution. To capture some variation in grazing intensity and the intensity of livestock keeping as such, we included estimates on the average density of ruminant (cattle, buffalo, goats, sheep) and other livestock (pigs, chicken, ducks and horses) which we obtained from the latest global livestock density data [42]. We used the area-weighted (AW) version of the data from Gilbert et al. to reduce circularities due to different land cover products. Predicted counts of livestock heads were converted into standardized livestock units (LSU) using region-specific conversion estimates [55]. We calculated the average density of LSU—relative to total area in a target 5km grid cell—to be further considered in the analysis. We note that the percentage of animals that are actually grazing will vary by region and is context specific, thus these estimates are only an approximation of area use by animals.

Besides the final variables considered for this analysis, a much larger set of potentially relevant variables was originally considered. However, we removed many of those variables from the clustering based on expert feedback (S1 Fig in S1 File), high collinearity with other included dataset, undesirable data gaps or spatial artefacts or circularity of input datasets (some layers were for instance the result of statistical predictions based on different land cover reference datasets). Some of the variables that we decided not put into the global clustering, we considered separately in an overlay analysis (see section below). A list of all included variables for identifying the foodscape classes is highlighted in the supplementary materials (S1 Table in S1 File).

For the purpose of this analysis, we were only interested in identifying and clustering food producing regions. After consultation with experts and multiple iterations we decided to apply a series of filters on the 5km grid cells of level 1 and level 2. We only considered not water covered grid cells and also only land areas north of -60° latitude including Antarctica. Using the Copernicus land cover dataset we removed grid cells with highly urbanized land, identified through the upper quantile of the fraction of built up land (> 80%), from the analysis given that those areas are unlikely to be significant food production regions. We also removed areas with permanent snow cover (> 75%), hyper arid or polar deserts with bare land only (> 90%), e.g. BW or EF climatic zones according to [49] or grid cells with permanent water occurrence (equal to 100%) according to the fractional layers of the Copernicus land cover dataset [38]. Forested landscapes that are considered to be largely intact according to the Intact Forest landscapes layer [56] as well as grid cells with more than 95% of tree cover were also excluded, owing to the fact that those cells are unlikely to be significantly farmed at a broad 5km grid cell. The management related variables (level 2) relate to cropland and pasture lands only and we therefore removed from the level 2 clustering all grid cells that were not potentially grazed or cultivated, e.g. cells without any kind of fractional cover of bare, grass, crop or shrubland. We do acknowledge that many of the filtering thresholds are based on subjective decisions, although they were chosen based on multiple iterations, expert consultation and visual inspections of the resulting maps.

### Statistical analysis

All prepared variables were subjected to an unsupervised clustering algorithm so that areas with commonalities are grouped together, resulting in clusters that are formed based on spatial similarities in biophysical or management related variables. Here we used a neural-network clustering algorithm called self-organizing map (SOM), that is particularly suited for non-linear and heterogeneous data [57, 58]. Other, supervised clustering methods exist and can be the preferred method of choice when a-priori knowledge about the type and name of clusters exist [22]. However, the purpose of this work was to discover and let the data speak about emergent clusters using available global data. SOMs are able to reduce heterogeneous multi-dimensional sets of variables to a lower, two-dimensional space where topological properties are preserved among neighbours, making them ideal for the visual exploration and clustering of geographic data, such as data on food production [25, 58]. We conducted all procedures and clustering separately for each variable grouping (biophysical lvl1 and management lvl2, S1 Fig in S1 File). The rationale behind this approach was to support an easier labelling and ability to separate biophysical and management components, while still resulting in an intermediate level of complexity for classes. Most variables in the SOM were continuous with exception of soil type which we included as a binary variables. Although binary variables can have a considerable influence on the resulting clusters, we believe it is justified given the influence soil has on any kind of crop production.

A key decision in SOM parametrization is the resulting node size, which directly influences the number of classes obtained. Choosing an optimal node size is commonly a tradeoff between maximizing node quality, separability in terms of variance retained and the occurrence of imbalances or an excess of empty nodes. To identify an optimal node or cluster size, we performed a sensitivity analysis with varying node sizes ranging from 2x2, 3x2 up to 15x10 possible clusters. For each we build rectangular grids of nodes with 1.5 x times the width compared to height and for computational efficiency we extract (random sampling) values from all variables equivalent to 50% of the total grid size. We then performed a SOM clustering on each subset and calculated the mean distance between nodes, the average node quality, the Davies-Bouldin cluster index that measures intra- and inter- variability among nodes [59] calculated using the ‘clusterSim’ package [60] and the proportion of empty nodes as indication of separability. The optimal node size was then determined (see S2 Fig in S1 File for an example) through a joint minimization of the *Davies-Bouldin* cluster index and the break point in the mean distance between nodes while taking consideration of node quality and proportion of empty nodes [61].

To determine the most optimal allocation to clusters, further hyperparameter tuning can be beneficial. Other important hyperparameters to choose in unsupervised SOM clustering are those that determine size, number and stopping rules of the algorithm. For the SOM we tested a range of different parameters, altering learning rates, epochs, neighborhood sizes and distance decay for a hypothetical clustering of a 2x3 neural network. Based on a visual assessment of node quality we choose a starting learning rate of 1 and a finishing learning rate of 0.001, an epoch size of 50 as well default gaussian kernels with linear decay. Throughout we used a Queen’s case of a neighborhood function (3x3) as well toroidal grids, meaning that opposite edges of the reduced two-dimensional space of the SOM share their neighbouring nodes.

Although SOMs are the preferred method for the kind of spatial data used in this work, applying them at global scale can be quite costly in terms of computation time and resources as runtime scales exponentially with the number of grid cells. To perform the global clustering we used a high performance port of the popular Kohonen framework called Somuclu [62]. All other data preparation analysis was conducted in R [63], with occasional aggregation and exporting of data (particularly for Satellite-based data) being done with Google Earth Engine [64].

The maps of terrestrial classes at level 1 and level 2 were finally evaluated and further processed based on an expert consultation (S1 Fig in S1 File). SOMs partition the input datasets into classes within emergent properties and of similar variable loadings. Particularly in cases where the number of classes is large, a ‘post-hoc’ clustering of the SOM nodes is often desirable and possesses particular efficient properties compared to individual separate clustering algorithms [65]. We therefore further reduced the number of nodes by applying a k-means clustering on the codebook vectors obtained from the SOM [65]. Here the optimal number of clusters for k-means was determined using the ‘NbClust’ package across all possible indicators [66] and was bounded by the minimum dimension mapped by the SOM and the maximum possible combinations of a reduced set of clusters (N-1). We furthermore applied a modest global modal filter using a 3x3 moving window on each individual map to reduce minor ‘Salt-and-pepper noise’ resulting from inconsistencies and minor errors in mapped classes.

We note that although these measures taken post hoc necessarily result in a loss of detail, (a) they more closely refine an essential set of global clusters of food production making labeling and interpretation more viable, (b) clusters of both level 1 and level 2 were ultimately combined across levels (see below) thus combining strengths of both biophysical and management data in mapping foodscapes in a hierarchical manner [58], and (c) all clusters were carefully visually assessed by experts with knowledge of regional centers of food production and eventually further refined (i.e. by separating nodes from another) if deemed necessary. For example, based on a visual assessment of one class formed in level 2 as part of the post-hoc refinement, we decided to implement a further split up into two separate nodes in the SOM. Here we used the data on highly dominant irrigation (>75% of crops irrigated) and high nutrient application rates (>75% relative to global maximum) to separate out areas with these levels of intensity. These thresholds were chosen based on a visual assessment of the mapped variables and desirable features to be represented in the map.

### Visualization and labelling

The clustering conducted in this study does not follow any *a priori* agreed legend and there is thus a necessity to label each of the resulting clusters. The labelling was conducted as part of an expert assessment among co-authors and colleagues from regional representatives from the Nature Conservancy (TNC), where labels were agreed upon based on the variable loadings for each cluster and alignment between geographic distribution and regional knowledge. The labels were in a first iteration assigned by the author and expanded global TNC team, and then afterwards representatives of regional TNC teams were asked for their whether spatial delineation and name aligned with their knowledge of a region. Such an expert-derived labeling is common in interpretations of unsupervised clustering exercises [20, 23, 25]. To visualize the magnitude and direction of impact of each variable we extracted mean estimates of the scaled variables (see above, commonly referred to as z-scores) separately for each cluster. Positive z-scores refer to above-average, negative z-scores to below-average values regarding the indicator’s overall global mean.

In total we selected 11 classes at Level 1 and 10 classes at Level 2 for the terrestrial realm. Class labels at level 1 were multiplied with XX00 counting upwards and classes at level 2 with an index from 000X counting upwards to 00XX, thus enabling simple matching and overlap analyses. A draft combined map was then produced by overlaying the terrestrial classes at level 1 and level 2 and again subjected them to an expert assessment of the combined classes (S1 Fig in S1 File), after which we decided to merge a number of classes and decide on a taxonomy for the class labels. The terrestrial label taxonomy is based on a combination of predominant soil type, topography, land cover with varying descriptions of intensity and/or diversity of use as well as eventual grazing and other land cover. The final foodscape map contained a total of 86 labeled terrestrial classes.

For visualization all maps were reprojected into world Mollweide projection. For visualization convenience we also grouped all classes into three levels of intensity, namely “Scattered Cropland and grazing”, “Mixed and diverse food cultivation” and “Irrigated and/or intensive food production”. Flows between variables and production systems are visualized in a Sankey diagram with key elements of food production, such as the soil type, management intensity and type of crop are highlighted. All produced maps are made openly available as part of this manuscript (see data availability section) and can also be interactively explored and summarized online (https://www.globalfoodscapes.org/).

### Overlay analysis

The combined terrestrial foodscape map was finally overlaid with a series of 8 independent spatial layers to estimate to what extent foodscapes globally are exposed to pressure factors and biodiversity values. The layers considered in this overlay analysis are thus explicitly characterizing factors that affect global food production but are not necessarily part of the foodscape as such. First, we identified areas with crop- and grassland gain in the period 1992 to 2015 as ‘frontier expansion zones’ using data from the ESA CCI program [67, 68]. Data on the observed relative change in human population counts between 1975 to 2015 were calculated from the GHS POP dataset [69] and converted into a mask to reflect the areas with the 95% greatest population increase. To capture areas with declining yield trends across the world, we used reported spatial-explicit changes of yield estimates from the period 1961–2008 [70]. For the purpose of our overlay, we focused again only on areas with the largest yield losses (5%). Other important pressures on agricultural land are climatic variability, water depletion from irrigation water withdrawal, and soil erosion. We took data on global topsoil loss owing to water erosion from the Revised Universal Soil Loss Equation (RUSLE) model [71] and focused particularly on those areas with the greatest (95%) estimated topsoil loss. We also created a mask indicating areas that have an annual global depletion of more or equal than 75% or available water, using data from the WaterGAP model [72]. Climatic variability was estimated from an upcoming data layer of global climate hazards [73], where we particularly focused on areas with relative drought risk and/or high climate variability. Another considerable pressure on food production is the yield loss owing to crop pests. In the absence of any global spatial estimates of the pest-attributed loss in crop yield, we used downscaled global data of pesticide pollution risks [74], of which we considered the regions with the largest reported risks (95%). Finally, we also used a global mask of climatic zones from the Köppen-Geiger stratification to highlight present day climate [49].

To estimate exposure of foodscapes to global values related to biodiversity, we used spatial information from a new global priority map (ranked values 1–100) depicting areas with the greatest conservation value, e.g., those that contribute the most in reducing species extinction risk [75]. From this layer we created a mask of the 10% most valuable area for species conservation globally. In addition, we used data from TNC global Last chance ecosystem layers [76] which indicate the ecosystems most at risk on a scale from vulnerable, endangered to critical from human pressures globally, while considering current protection measures. Unless otherwise noted, we used data on the distribution of critical ecosystems for any overlay analyses. Lastly, we included a mask indicating areas with high numbers (95% quantile) of assessed threatened amphibian, bird and mammal species using data from the IUCN redlist (https://www.iucnredlist.org/resources/other-spatial-downloads). It should be stressed that the distribution of both the Last Chance ecosystem and the threatened species mask are not informed by the distribution of land cover, in contrast to the conservation priority map which is consistent with Copernicus land cover.

All layers were aligned—and resampled if differing in resolution—using nearest neighbour sampling to the global terrestrial foodscapes layer. Continuous layers were first scaled (mean subtracted and divided by standard deviation) and then summarized as 5% largest (95% percentile), or 5% smallest where noted, areas globally. Finally, we extracted the amount of intersecting foodscape area for each overlay. Overlays were explored by investigating the proportion of foodscape area exposed to a certain pressure or value. We used relative area measures because both the size of overlay masks and areas of foodscapes classes differ from each other. A stacked map of the global pressures and values can be found in S3 Fig (S1 File).

## Results

### State and distribution

**Fig 1:**
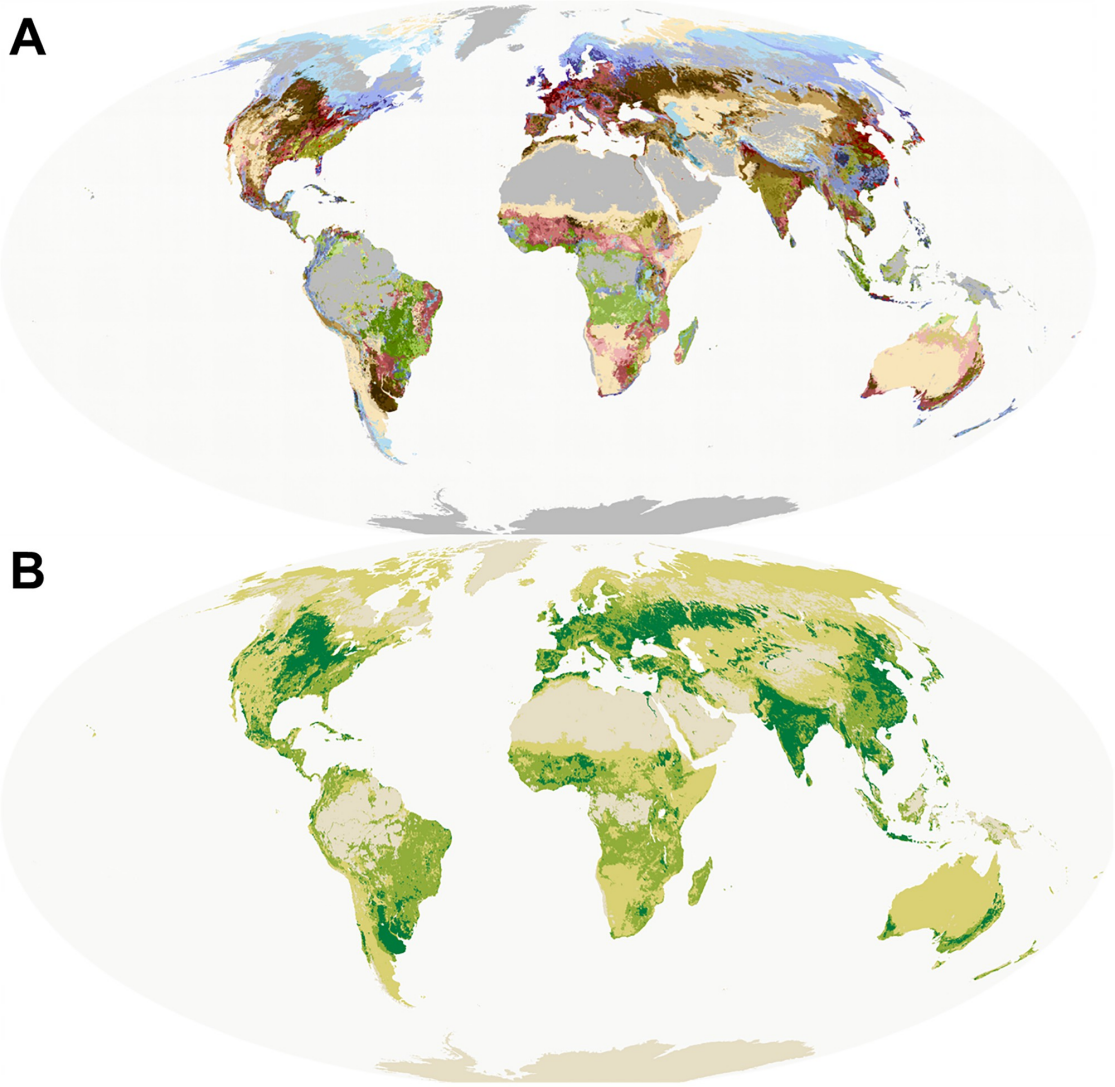
(A) The global foodscape map (combined for lvl1 and lvl2) for the terrestrial realm, (B) a layer where all foodscapes classes are remapped into intensities of use. Owing to the large number of classes a legend is not shown. A crosswalk between the intensity groupings in (B) and the foodscape classes can be found in S5 Table (S1 File). Owing to the large number of classes a legend is not displayed, however class descriptions and labels can be found in S3 Table (S1 File).

Humans produce substantial amounts of food in the form of farming and grazing globally. We identified that broader landscapes containing food production comprise 63% (9113 million ha) of the terrestrial land area with the remaining area only under marginal to little food production. We identified a total of 86 terrestrial foodscapes classes globally that are distinct in various aspects of food production and management (Fig 1). The average foodscape class has a size of about 168 million ha (∓ 514.85 SD). The most spacious terrestrial foodscape class is ‘*Entisols on plains with grazed bare land and grass cover*’ and covers 9.3% of global land area (1341 million hectare), while the smallest terrestrial foodscape class ‘*Oxisols on humid tree-covered land with little food production*’ covers just 0.005% (0.72 million ha) of terrestrial land area (S3 Table in S1 File). When recategorized into intensity of use, we find that approximately 34.5% (4988 million ha) of the terrestrial land surface are only under scattered cultivation and grazing, 21% (3113 million ha) are under mixed and diverse, and 12% (1721 million ha) under irrigated and/or intensive production (Fig 1B). As also stressed in the discussion all these estimates are conditional on the underlying data used and our classification of intensities.

Different types of foodscapes classes are spatially clustered globally and some parts of the world harbor regions with on average greater heterogeneity than others. Tropical and dry climatic zones of the world contain the largest number of terrestrial foodscapes classes, with several climatic zones being dominated by intensively cultivated foodscapes classes (S4 Table in S1 File). Critically, foodscapes classes labelled as intensively cultivated are already the most predominant in 5 of the 30 terrestrial climate zones of the world, with the other climatic zones having foodscapes classes labeled as diverse and often used by smallholders (17) or little to no food production (8) as the most dominant. The most common foodscape class on the South American continent was “*Oxisols and Ultisols with mixed grazing and crop production on large fields*”, while in North America and Europe foodscapes classes identified as scattered food production in mixed forests, grasslands and grazed land was the most common.

**Fig 2:**
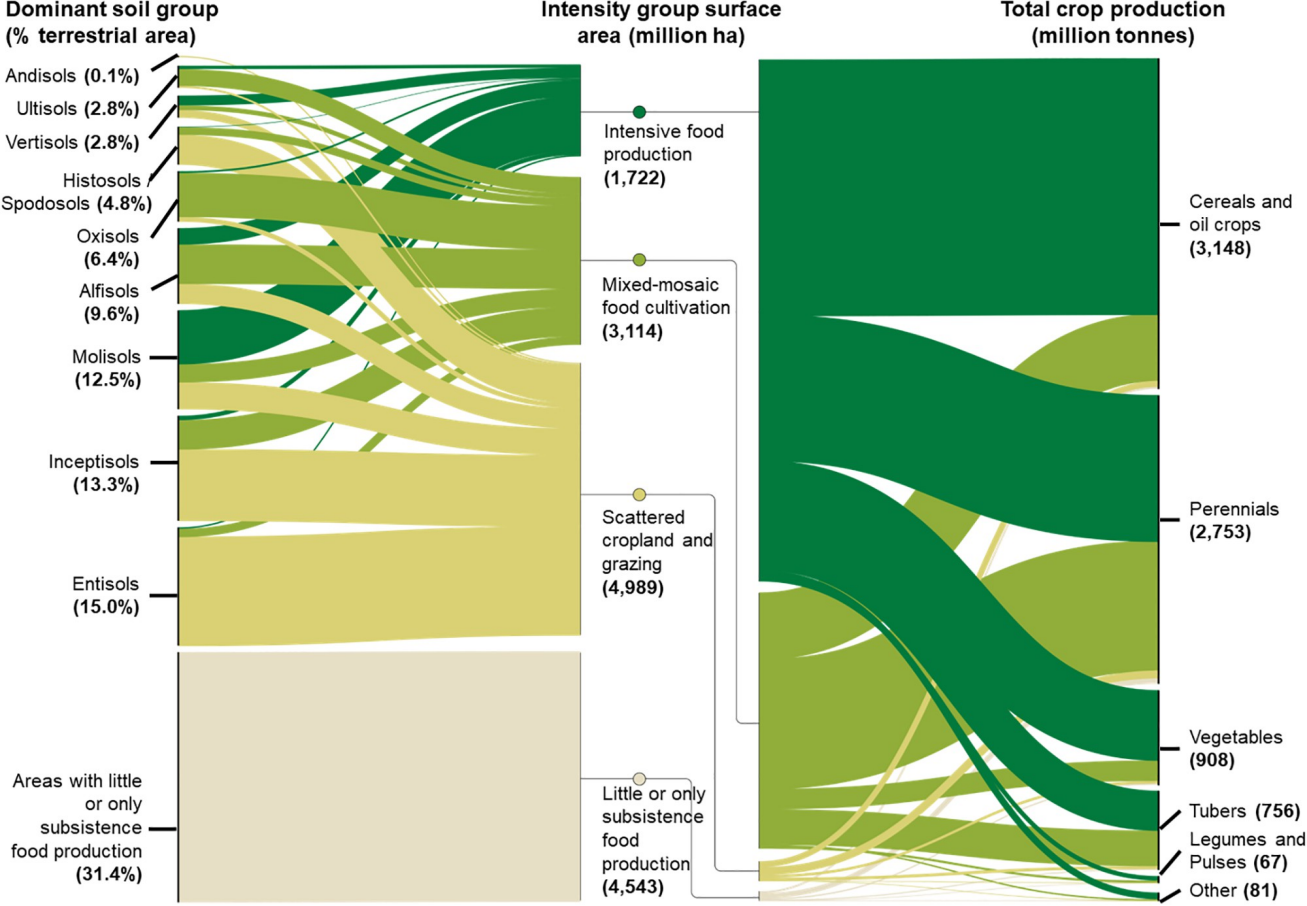
Sankey diagrams highlighting the shares of dominant soil type, the intensity of cultivation and total crop production across all foodscape classes. Values indicate the percent of terrestrial land area, million ha and million tons respectively. Intensity group colors as in Fig 1B. See S3 Table in S1 File for a legend and crosswalk table between foodscape classes and intensity groups. Figure adapted from a version created by Nicholas Rapp for the TNC Foodscape report [77].

Out of the total 9,824. million ha of land that contains more than marginal food cultivation, and producing cereals, tubers, vegetables and other crops, 16.2% (1,722 million ha) is under intense food production, compared to the 36.9% (3,630) of mixed-mosaic and 46% (4,601) of scattered land under cultivation and grazing (Fig 2). Yet, on the ~16.2% of intensively managed land, or the combined area of 26 different foodscape classes, about 64.5% of all cultivated crops for human or animal consumption are grown (Fig 2), indicating the high dependency of food production to a select few regions. Cereals and oil crops from the largest part (41%) of all produced crops and are almost exclusively produced in foodscape classes with intensive or mixed-mosaic food production (Fig 2). Interestingly, almost half of all perennial crops and tubers are grown in mixed-mosaic food production systems (Fig 2), highlighting the importance of complex landscapes for the global food sector. All soil profile groups were present across the foodscape classes with Molisols being most intensively cultivated and Entisols the least despite being the most common (Fig 2). Overall, these statistics highlight both diversity and concentration of food production within the mapped foodscape classes.

### Pressure exposure

**Fig 3:**
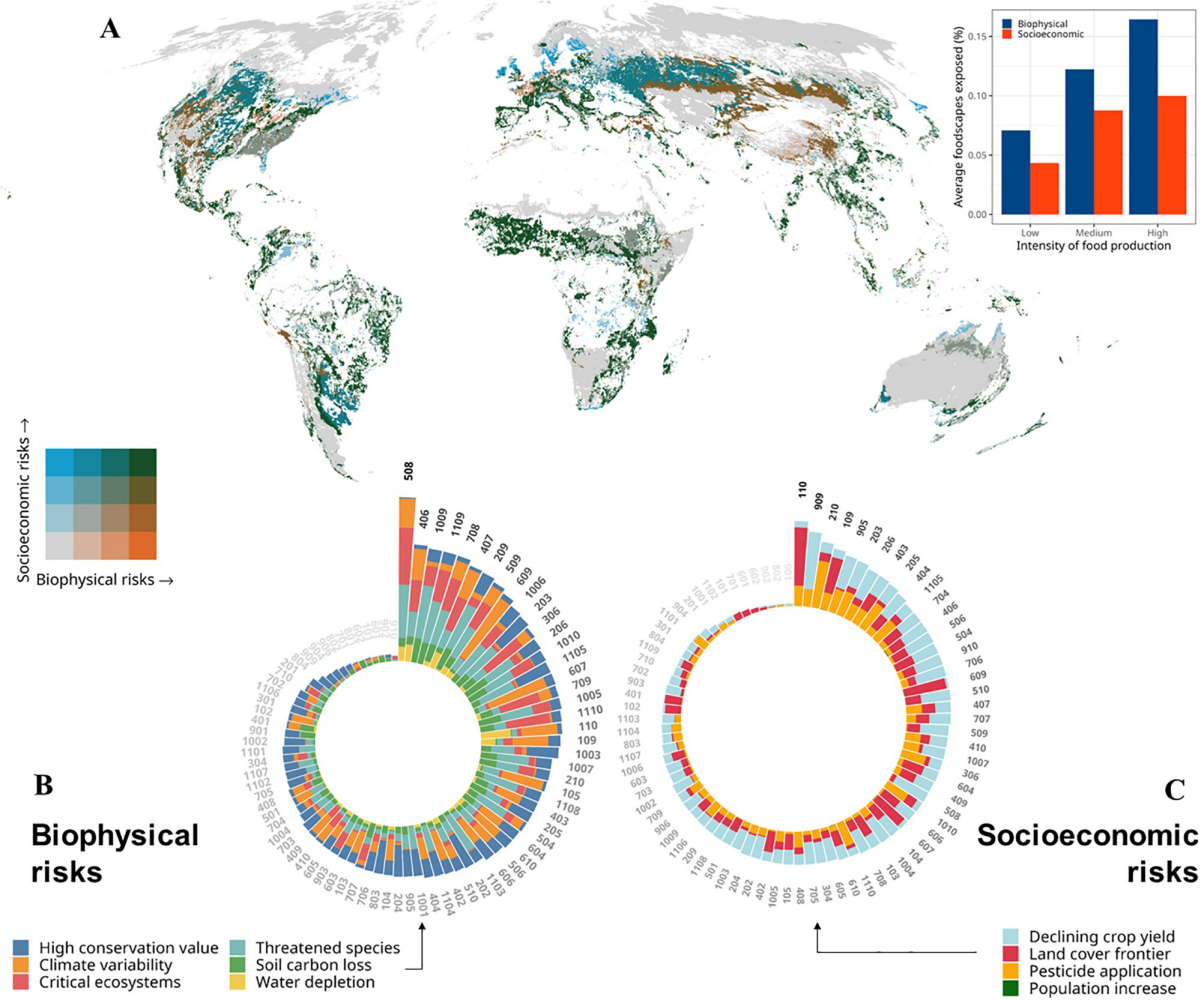
Intersection of foodscapes area with biophysical and socioeconomic risks globally (A). Shown on a bivariate scale are those foodscapes most exposed by single or multiple pressures, with darker green colours indicating a greater exposed proportion of the individual foodscape (Fig 1). Barplot highlights the average proportion of exposed foodscape to biophysical or socio-economic risks. Circular plots show cumulative exposed proportion of foodscape area by pressures separated by biophysical (B) and socio-economic risks (C).

Many of the mapped foodscape classes are affected by a series of pressures and risks, while also being in regions considered to be of high value to biodiversity. In terms of absolute area, food production in grazed Entisols on bare land (class 102) is the most exposed to high climate variability (18 mill. ha) while being situated in regions of expanding agriculture and pasture (13.7 mill. ha). Almost half of the total area of food production in classes such as *“Inceptisols in arid hilly land with rainfed cereal and legume production and other livestock”* (610) or “*Entisols on dry rainfed plains with legumes and pulses production and occasionally other crops*” (110) were affected by high climate variability and particularly noteworthy is also a group of foodscapes classes (607, 607, 610, Fig 3A) each with similar properties, being intensively irrigated and on Inceptisols in often arid and hilly land. These data highlight the increasing vulnerability of food production in dry cropland and rangelands systems and in some of the most intensively cultivated food production regions.

Most foodscapes classes are not only exposed to single, but multiple pressures globally (S3 Fig in S1 File). On average 39.4% (range 1.7% to 92.2%) of the terrestrial foodscape area is exposed to at least one considered pressure variable. Critically, foodscapes classes considered to be irrigated and/or intensive cultivation (Fig 1B) were more often (33.7%) than less intensively cultivated foodscapes (scattered 21.2% and mixed use 26.4%) affected by more than one pressure. Among those foodscapes classes dry bare and grazed land dominated by Entisols (classes 102 & 103) as well as intensively cultivated Mollisol foodscapes (i.e. classes 409 & 402) are particularly exposed to multiple pressures such as climate variability or water depletion (Fig 3). Most foodscape classes that are exposed to high climatic variability tend to be in regions that can be considered as land cover frontier, depleted of groundwater and with substantial increases in human population density (Fig 3), bringing food production in those regions at risk. Crop production in foodscape classes with high topsoil loss and often substantial pesticide application were more often in regions that are of high importance to threatened species and can be considered critical ecosystems (Fig 3).

Foodscapes that more exposed to biophysical risks also tend to be more exposed to socio-economic risks (r = 0.482, df = 83, p < 0.001). The most common combination of co-occurring biophysical pressures on foodscapes classes were high climate variability and severe water depletion, particularly in areas with a recent expansion of agriculture and pastures (average proportion of all foodscapes 39.3%) or reduction in crop yield (27.7%). Crucially, foodscape classes that contained critical ecosystems or high amounts of threatened species were also exposed to considerable climate variability (37.2%) or topsoil loss (34.4%). These combinations of risks with biodiversity seem to occur more often than others, potentially indicating that some of the main pressures to food production are in areas where species are either threatened or where potential conflicts between food production and biodiversity might arise.

Indeed, food production in many parts of the world takes place in regions that are of high biodiversity conservation value or contain critical ecosystems. On average 34.3% (range 0.3% to 86.1%) of the area of foodscapes can be considered of some value to biodiversity (Fig 3). The foodscape class that had most of its area intersecting with the distribution of critical ecosystems and threatened species are mixed production landscapes predominantly distributed in India (class 508), while notably mountainous tropical and south-east Asian forested areas had high conservation value (Fig 3), with Foodscape classes such as *Inceptisols on humid hilly-mountains with tree cover and small farmed mixed and intensive diverse production* (class 203) containing the most critical areas in terms of extinction risk reduction and threatened species, highlighting the conservation value of mixed and diverse small-holder production systems in tropical countries.

## Discussion

In this work we identify and map global classes of analogous centers of food production, or foodscapes. There are 86 distinct foodscape classes of broadly comparable biophysical and socio-economic conditions (Fig 1), some of which can only be found in certain geographic regions (S4 Table in S1 File), while others are distributed across all realms. We find that intensively cultivated foodscapes classes in terms of total area are already the most dominant in 5 of the 30 terrestrial climatic zones of the world, although foodscapes with mixed purposes were most common falling into the areas shared between natural land cover and human modified land [78]. Further, we find that global food production was highly concentrated in a few foodscapes classes, with about 60% of all global food being produced on about 16.2% of terrestrial land area (Figs 1 and 2). Yet our results also indicate that not all foodscape classes are equally responsible for crop production globally and thus discussions about sustainable intensification or management interventions would benefit from a global and regional perspectives such as the one provided by the concept of foodscapes.

A more optimised food production by crops in some foodscape classes could contribute to a more sustainable food system and align production needs and constraints with those of regional and global agendas. Despite most cereal and oil crops being produced in few foodscape classes, we also found that a substantial amounts of perennial, tuber and vegetable production occurs in foodscape classes considered to be of mixed landscapes (Fig 2). This indicates such landscapes might require more integrated solutions and interventions that consider multiple objectives [79, 80]. Especially since many of those crops types are deemed to be key importance to healthy diets globally [6, 8]. Further, some of the most productive groups of soils, e.g. Mollisols, Alfisols and Oxisols, contain a large share of land with potentially inefficient production methods compared to other land in the same foodscape classes (Fig 2). Although we only visually investigated broad patterns of crop production (Figs 1 and 2), ignoring other key constraints such as market access or potential crop suitability [15], this might indicate that there exist opportunities to sustainably intensify and manage foodscapes better in a regional and global context [77]. Potentially NBS, such as restoration, agroforestry or better soil, watershed and nutrient management can effectively address inter-related challenges to help humanity stay within its planet boundaries [81, 82]. Overall, the concept and our initial mapping of foodscape can contribute towards identifying opportunities for agri-economic interventions and sustainable intensification, especially in areas with inefficient current production or with considerable risk factors.

The spatial concentration of global food production in very few foodscape classes also results in considerable risks towards food security [83] as well as creating tensions with the achievement of other sustainable development goals such as clean water or life on land [81, 84]. We also find that about ~40% of all foodscape classes are exposed to single or multiple pressures globally (Fig 3), with particularly intensively cultivated foodscapes being more often affected by multiple rather than single pressures. Notably foodscapes exposed to high climate variability and water depletion are also more likely to be situated in an area considered as a frontier zone (e.g. land with expanding cropland or grassland), which could indicate that intensifying production in these areas might not be sustainable in terms of food production. However, there are many theories behind what drives land-cover and land-use change at the agricultural frontier [85, 86], and depending on the assumptions behind the expansion of a frontier it could be seen not as pressure on food production, but a driver and key factor in the creation of novel foodscape classes [87].

The finding that foodscapes classes with high soil erosion and pesticide inputs often co-occur (Fig 3), highlights the need to address possible interacting effects [81], for example, increased risk of pesticide pollution in water bodies owing to runoff caused by soil erosion and unsustainable agricultural production [88]. Finding possible interventions for such interacting pressures might be one way to address multiple aligned pressures [80], and maps of foodscapes could be considered as possible decision support tool to reveal homologous areas in which similar interventions and strategies could be applied.

Our results also strikingly reveal that the diversity of wild animals and plants on Earth is in many places interlinked with different types and intensities of food production, with tree covered and small-holder dominated foodscapes commonly covering areas with high biodiversity value (Fig 3). Possibly those species are able to persist in smaller habitats within these foodscapes, or they have low sensitivity to pressures related to agriculture and occupy the same space (“land sharing”, [11]). It could also be that biodiversity-mediated benefits for crop production may be at peril in the same regions as those services usually are similarly affected by landscape simplifications, climate variability and soil degradation [89], which may reinforce losses in crop productivity. Overall, this emphasizes the need to find integrated solutions to the biodiversity-food nexus [7, 79], so as not to jeopardize the value of local food production biodiversity and increase food security risks.

### Error sources and caveats

Similar to most analyses at global scale there are a number of caveats and limitations, and we stress that the current map of foodscape classes is a global assessment and does not intend or can reflect the full diversity of foodscapes at local or sub-national scale. Notably we do not claim to have mapped the entire “foodscape” comprehensively, only distinct classes that can be used to identify broad-scale synergies and homologous situations [90]. Thus, when using the foodscape map in subsequent analyses it is important to be aware of a number of error sources and caveats, including spatial, temporal and thematic mismatches:

**Spatial** Some of the delineations of foodscape classes can be inaccurate owing to mismatches in spatial resolution of the input layers, where some layers are only available at a resolution coarser than the target 5 km grid cell. For the analysis we disaggregated those datasets to a finer scale, which might cause not only spatial artefacts, but can also misrepresent the target variable values. Similarly, by aggregating fine-scale data (such as global 100 m Copernicus land cover data) to a coarser scale there is a loss of detail and information. We believe these mismatches in resolution are acceptable given the global scale of our analyses, however stress that cluster boundaries should be interpreted with caution. Similarly, It should be highlighted that some of the management related data layers are downscaled census data at regional or national scale, which can cause spatial artefacts due to the Modifiable Areal Unit Problem (MAUP). This might also relate to an overestimation of certain management practices, for instance, broad areas with little cropland cover can be classified as having high nutrient input if data for nutrient application rates was derived from regional rather than farm level. **Temporal** There can also be mismatches in temporal resolution. Our work broadly focused on the time span between 2010 and 2015 and a number of the used datasets were produced years ago. Land management practices, however, are dynamic and often change between year [91], there can be temporal mismatches between different input variables and thus identified clusters. We partly account for this uncertainty by merging smaller clusters and a posthoc reduction of speckle. **Thematic** Many of the included variables rely—also indirectly through auxiliary data used to create those variables—on different definitions, for example of what constitutes for instance a cropland or pastoral system. We accounted to some degree for this issue by pre-filtering grid cells for management related variables to only those with significant proportions of crop or grassland (see Methods) and by relying on Copernicus land cover data where possible [38].

### Gaps and missing data

In this work we used some of the best available global data on biophysical and management related factors of the food sector, fully recognizing that some data simply do not yet exist at global extent or at the spatial resolution that would allow a delineation of foodscapes according to our definition. For example, we lack higher resolution and temporally up to date data on pastoral grazing intensity, farm size (opposed to field size) or thematically well resolved systems of agroforestry types (e.g. intercropped systems opposed to home-garden systems). Similarly in our overlay analysis we did not consider factors related to the genetic diversity of crops owing to a lack of openly available at a sufficient resolution [92] and also ignored biodiversity mediated factors related to crop production, such as pollination or pest-control [89]. Lastly it should be highlighted that there are inherent uncertainties in all spatial products, and that such errors can naturally propagate into analyses such as ours [93]. We highlight the need for more highly resolved, recent and accurate global data on land use and management practices.

## Conclusion

The concept of foodscapes enables a more holistic view towards food production, enables the planning of interventions across similar homologous regions and help the design of more integrated planning approaches [94, 95]. For example, it can serve as a starting point to identify benefits of nature-based solutions that benefit the climate, contribute towards more sustainable agriculture and also free up land for restoration [77]. In particular the identification of opportunities for ecosystem restoration [96] together with solutions that maximize synergies and minimize tradeoffs with food production can provide a valuable avenue for further research [11, 79]. However, any restoration actions need to be undertaken with careful consideration of regional and local socio-economic factors as often the highest potential for restoration exists in areas with human inhabitation or intensively cultivated land [77, 96, 97]. Similar use cases might involve the prioritization of climate adaptation and mitigation actions in the face of climate change by evaluating synergies and tradeoffs with sustainable food production. In future work we plan to build on similar archetyping exercises at finer resolution, for example by building nested representations within broader foodscapes at national and regional scale, or by identifying bundles of transformation pathways within and across homologous regions together with stakeholders [28, 90]. Clearly, the mapping and identifying foodscapes at global scale might be only the start of the quest for a better representation of the diversity of food systems.

In this work we present a global stratification of food production systems or foodscapes, based on currently best available global biophysical and socio-economic data and following an intensive refining and expert-based assessment. We make these new maps of foodscape classes openly available at various levels (see data availability statement). We anticipate the newly created maps to be particularly useful for large-scale intervention, global modelling exercises and scenario-based analyses, or for the identification of homologous food production regions to conduct more detailed integrated planning at regional or national scale.

## Supporting information

S1 File Additional details and supplementary tables. Further spatial summaries and tabular description of the used input data can be found in the Supporting information.
(DOCX)
