# Supplementary material for: A global clustering of terrestrial food production systems
Source: PLoS One. 2024 Feb 14;19(2):e0296846. doi: 10.1371/journal.pone.0296846 (PMC10866528; doi:10.1371/journal.pone.0296846)
Supplement: S1 File — Further spatial summaries and tabular description of the used input data can be found in the Supporting information. (DOCX) [file pone.0296846.s001.docx]

Supplementary Information for

A global clustering of terrestrial food production systems

Jung *et al.*

*Corresponding author. Email: [jung@iiasa.ac.at](mailto:jung@iiasa.ac.at)

Fig. S1.

**
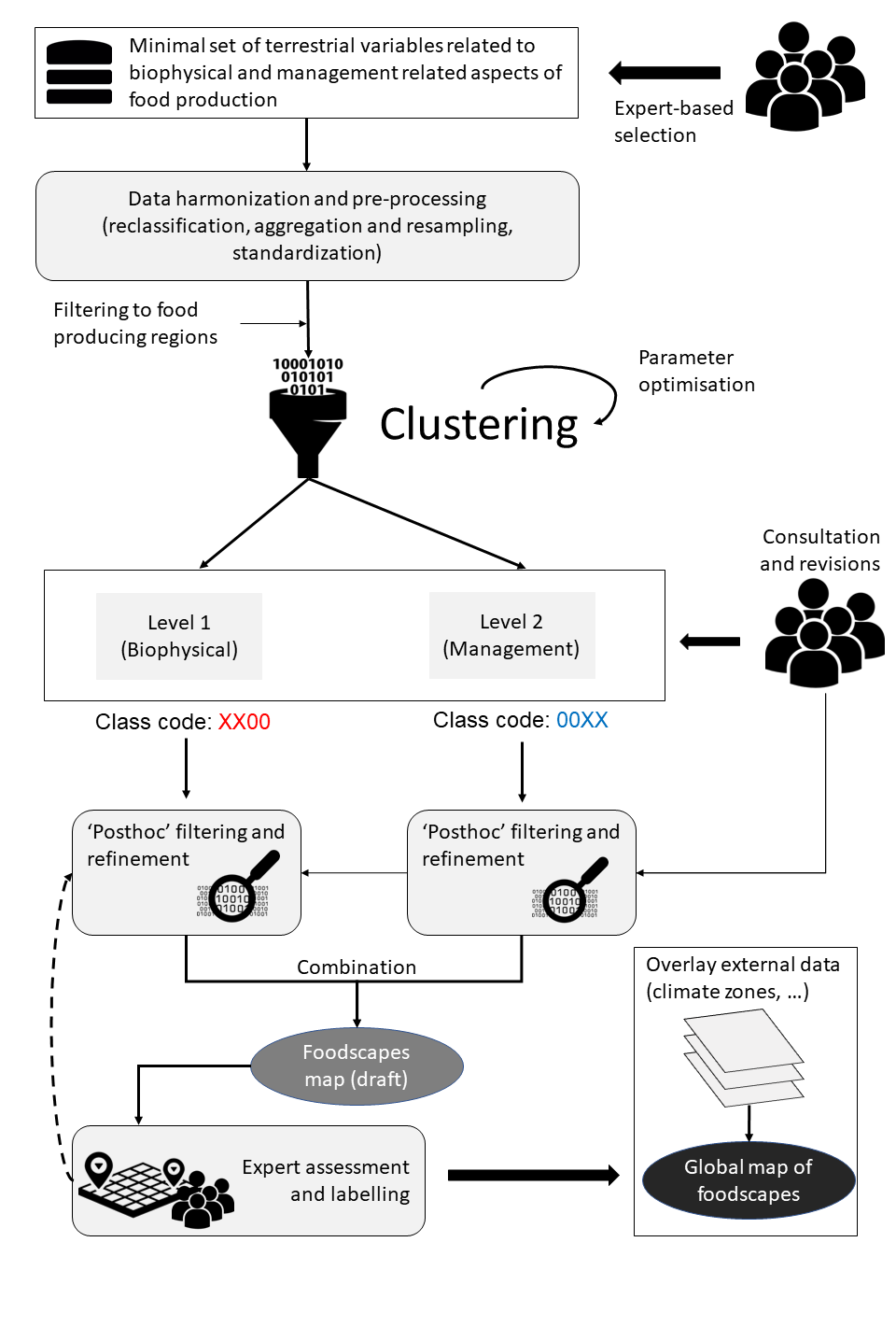
**

S1 Fig. **Schematic of the two-level clustering procedure to obtain the global foodscapes map**. Including critical consultation and revision steps. See Methods for more details.

Fig. S2


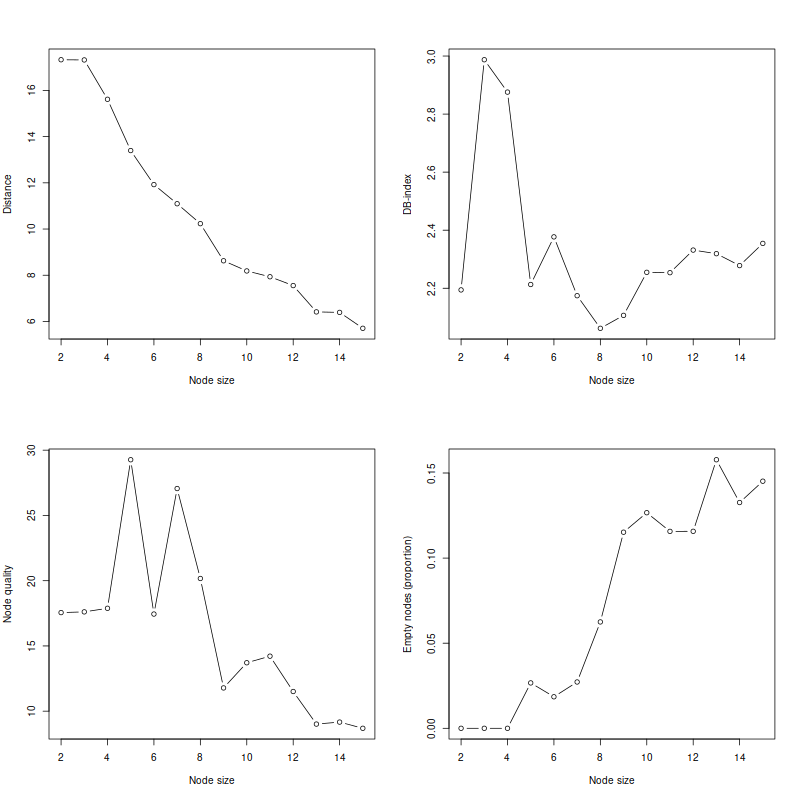


S2 Fig. **Example visual identification of an optimal clustering**. Showing the mean distance between nodes, the Davis-Bouldin Index, the average Node Quality and the proportion of empty nodes across the network. An optimal number of clusters for this example would be a total of 8 nodes to obtain a local minima of the Davies-Bouldin Index, while also obtaining optimal node quality and a minimal number of empty nodes.

Fig. S3


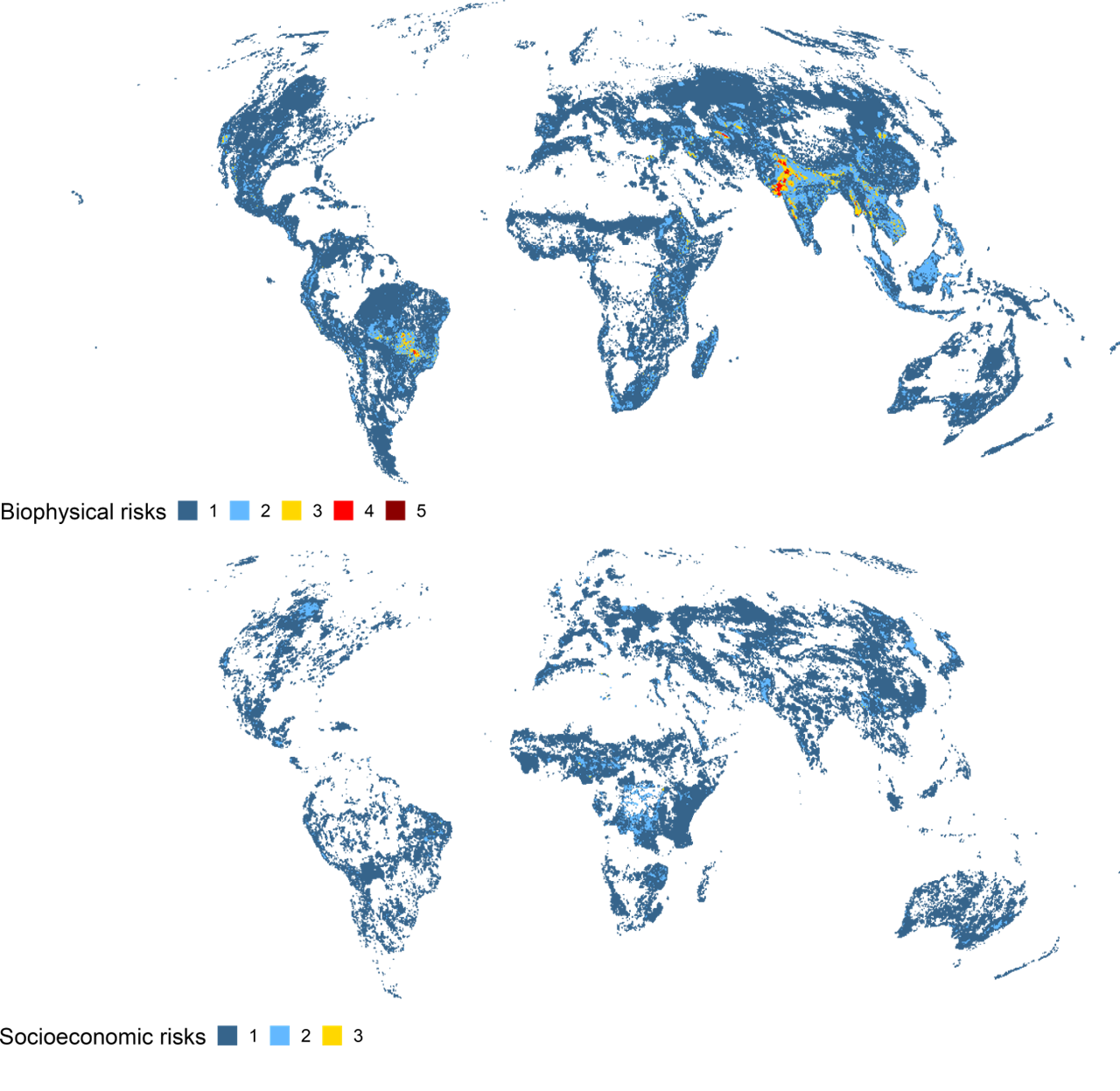


S3 Fig. **Stacked maps of the used overlays for both biophysical and socio-economic risks.** Overlay (sum) of selected biophysical and socio-economic risks.

S1 Table. **Included variables, rationale, unit, source and preprocessing requirements**. Provided are original data sources and acquisition links as well as a short description of the preprocessing steps undertaken.

| **lvl** | **variable** | **Description** | **Unit** | **Intention** | **Source** | **PreProcessing** |
| --- | --- | --- | --- | --- | --- | --- |
| 1 | Hills | Fractional amount of hilly terrain in an area | continuous | Get foodscapes on hills | <https://zenodo.org/record/1464846> | Calculated as fraction of specific terrain within a 5km grid cell |
| 1 | Mountains | Fractional amount of mountaineous terrain in an area | continuous | Get foodscapes on mountains | <https://zenodo.org/record/1464846> | Calculated as fraction of specific terrain within a 5km grid cell |
| 1 | Plains | Fractional amount of plains terrain in an area | continuous | Get foodscapes on plains | <https://zenodo.org/record/1464846> | Calculated as fraction of specific terrain within a 5km grid cell |
| 1 | usda_alfisols | USDA Alfisols dominant in grid cell | binary | To represent soil orders relevant for crop systems | <https://zenodo.org/record/3528062#.YBAkRtbTXRY> | Take predominant layer per group, reclassify to target USDA soil group and then modal aggregate from 250m to 5km |
| 1 | usda_andisols | USDA Andisols dominant in grid cell | binary | To represent soil orders relevant for crop systems | <https://zenodo.org/record/3528062#.YBAkRtbTXRY> | Take predominant layer per group, reclassify to target USDA soil group and then modal aggregate from 250m to 5km |
| 1 | usda_entisols | USDA Entisols dominant in grid cell | binary | To represent soil orders relevant for crop systems | <https://zenodo.org/record/3528062#.YBAkRtbTXRY> | Take predominant layer per group, reclassify to target USDA soil group and then modal aggregate from 250m to 5km |
| 1 | usda_histosols | USDA Histosols dominant in grid cell | binary | To represent soil orders relevant for crop systems | <https://zenodo.org/record/3528062#.YBAkRtbTXRY> | Take predominant layer per group, reclassify to target USDA soil group and then modal aggregate from 250m to 5km |
| 1 | usda_inceptisols | USDA Inceptisols dominant in grid cell | binary | To represent soil orders relevant for crop systems | <https://zenodo.org/record/3528062#.YBAkRtbTXRY> | Take predominant layer per group, reclassify to target USDA soil group and then modal aggregate from 250m to 5km |
| 1 | usda_mollisols | USDA Mollisols dominant in grid cell | binary | To represent soil orders relevant for crop systems | <https://zenodo.org/record/3528062#.YBAkRtbTXRY> | Take predominant layer per group, reclassify to target USDA soil group and then modal aggregate from 250m to 5km |
| 1 | usda_oxisols | USDA Oxisols dominant in grid cell | binary | To represent soil orders relevant for crop systems | <https://zenodo.org/record/3528062#.YBAkRtbTXRY> | Take predominant layer per group, reclassify to target USDA soil group and then modal aggregate from 250m to 5km |
| 1 | usda_spodosols | USDA Spodosols dominant in grid cell | binary | To represent soil orders relevant for crop systems | <https://zenodo.org/record/3528062#.YBAkRtbTXRY> | Take predominant layer per group, reclassify to target USDA soil group and then modal aggregate from 250m to 5km |
| 1 | usda_ultisols | USDA Ultisols dominant in grid cell | binary | To represent soil orders relevant for crop systems | <https://zenodo.org/record/3528062#.YBAkRtbTXRY> | Take predominant layer per group, reclassify to target USDA soil group and then modal aggregate from 250m to 5km |
| 1 | usda_vertisols | USDA Vertisols dominant in grid cell | binary | To represent soil orders relevant for crop systems | <https://zenodo.org/record/3528062#.YBAkRtbTXRY> | Take predominant layer per group, reclassify to target USDA soil group and then modal aggregate from 250m to 5km |
| 1 | Tree | Fractional amount of tree cover in a given area | continuous | Cover variation of land cover per class | <https://lcviewer.vito.be/> | Aggregated (average) from native 100m to 5km resolution |
| 1 | Crops | Fractional amount of crop cover in a given area | continuous | Cover variation of land cover per class | <https://lcviewer.vito.be/> | Aggregated (average) from native 100m to 5km resolution |
| 1 | Grass | Fractional amount of grass cover in a given area | continuous | Cover variation of land cover per class | <https://lcviewer.vito.be/> | Aggregated (average) from native 100m to 5km resolution |
| 1 | Shrub | Fractional amount of shrub cover in a given area (note some shruby crops might be mixed in here according to the Copernicus technical document) | continuous | Cover variation of land cover per class | <https://lcviewer.vito.be/> | Aggregated (average) from native 100m to 5km resolution |
| 1 | BuiltUp | Fractional amount of builtup urban cover in a given area | continuous | Cover variation of land cover per class | <https://lcviewer.vito.be/> | Aggregated (average) from native 100m to 5km resolution |
| 1 | Bare | Fractional amount of bare cover in a given area | continuous | Cover variation of land cover per class | <https://lcviewer.vito.be/> | Aggregated (average) from native 100m to 5km resolution |
| 1 | agroforestry | Agroforestry mixed landscapes with tree cover as fraction of a grid cell | continuous | Helper for separating out mixed forest agricultural sites in the tropics | [unpublished yet, but some methods can be found here https://zenodo.org/record/3933966](https://zenodo.org/record/3933966) | Take grid cells that are of agroforestry type, e.g. crop production that involves significant tree cover. Fractional aggregate to 5km |
| 1 | aridity | Continious measure of aridity in a given area | continuous | Continious measure of aridity to capture variation of aridity to humidty within climate | <https://doi.org/10.6084/m9.figshare.7504448.v3> | Averaged for each 5km grid cell |
| 1 | lgp | Length of growing period | continuous | Indication of agricultural suitability | <http://www.fao.org/geonetwork/srv/en/resources.get?id=14057&fname=Map4_2.zip&access=private> | Dissaggregated to 5km resolution and classes with 365 days per year merged |
| 2 | area_singlecropping | Total harvest food with single cropping from Waha et al (MIRCA model) | continuous | Identify cultivated areas that single cropped | <https://doi.org/10.1016/j.gloenvcha.2020.102131> | Take both irrigated and rainfed portions, disaggregate them and resample them to target grid resolution of 5km |
| 2 | tillage_convrot | Prediction of tillage practices. Combined binary estimate of all conventional tillage practices | binary | Is cropping being predimonantly of conventional tillage | [Porwollik, Vera; Rolinski, Susanne; Müller, Christoph (2019): A global gridded data set on tillage. V.1.1. GFZ Data Services. http://doi.org/10.5880/PIK.2019.009](http://doi.org/10.5880/PIK.2019.009) | Combine conventional and traditional annual tillage together , then assess the mode of this value per 5km grid cell |
| 2 | tillage_reducedCons | Prediction of tillage practices. Combined binary estimate of all reduced and conservation agriculture tillage practices | binary | Is cropping being predimonantly of reduced tillage and conservation agriculture | Porwollik, Vera; Rolinski, Susanne; Müller, Christoph (2019): A global gridded data set on tillage. V.1.1. GFZ Data Services. http://doi.org/10.5880/PIK.2019.009 | Combine reduced tillage and conservation agriculture together. Then assess the mode of this value per 5km grid cell |
| 2 | tillage_traditional | Prediction of tillage practices. Combined binary estimate of all traditional tillage practices | binary | Are traditional tillage practies being used? | Porwollik, Vera; Rolinski, Susanne; Müller, Christoph (2019): A global gridded data set on tillage. V.1.1. GFZ Data Services. http://doi.org/10.5880/PIK.2019.009 | Combine rotational and traditional rotational tillage together. Then assess the mode of this value per 5km grid cell |
| 2 | AAW_nutrientapplicationrate | Overall average area-weighted nutrient application in a given area | continuous | How strongly is an area fertilized | <http://www.earthstat.org/nutrient-application-major-crops/> | Calculate the average rate of application per crop and then weight by area. Windosized by 95% quantile and aligned bilinearly (mean) to 5km grid cell |
| 2 | area_cropdiversity | Diversity of production of crops according to SPAM | continuous | Diversity of production | [https://www.mapspam.info/data/ AND https://s3.amazonaws.com/mapspam/2010/v1.1/readme_v1r1_global.txt](https://s3.amazonaws.com/mapspam/2010/v1.1/readme_v1r1_global.txt) | Calculated as shannon index of all production estimates according to SPAM |
| 2 | spam_prodstaplecrop | Total production of staple crops, so those "maize","rice","wheat","potato","cassava", "soybean","sweet potato","yams","sorghum","plantain" | continuous | Useful for identifying the world's bread baskets | <https://www.mapspam.info/data/> | Take the total production value of all considered crops and aggregate them with a sum |
| 2 | spam_propCO | Proportion of cereals and oil crops grown in an area | continuous | Identify dominant type of crop group | [https://www.mapspam.info/data/ AND https://s3.amazonaws.com/mapspam/2010/v1.1/readme_v1r1_global.txt](https://s3.amazonaws.com/mapspam/2010/v1.1/readme_v1r1_global.txt) | Take the physical production area of all crops in a given grid cell and calculate the proportion their production makes up. Then determine the group of crops (see table) that is most predominant. Modal disaggregate to target 5km grid |
| 2 | spam_proprootstubers | Roots and tubers as proportion of crops grown in an area | continuous | Identify dominant type of crop group | [https://www.mapspam.info/data/ AND https://s3.amazonaws.com/mapspam/2010/v1.1/readme_v1r1_global.txt](https://s3.amazonaws.com/mapspam/2010/v1.1/readme_v1r1_global.txt) | Take the physical production area of all crops in a given grid cell and calculate the proportion their production makes up. Then determine the group of crops (see table) that is most predominant. Modal disaggregate to target 5km grid |
| 2 | spam_proplegumespulses | Legumes and pulses as proportion of crops grown in an area | continuous | Identify dominant type of crop group | [https://www.mapspam.info/data/ AND https://s3.amazonaws.com/mapspam/2010/v1.1/readme_v1r1_global.txt](https://s3.amazonaws.com/mapspam/2010/v1.1/readme_v1r1_global.txt) | Take the physical production area of all crops in a given grid cell and calculate the proportion their production makes up. Then determine the group of crops (see table) that is most predominant. Modal disaggregate to target 5km grid |
| 2 | spam_propperrenials | Perrenial crops as proportion of crops grown in an area | continuous | Identify dominant type of crop group | [https://www.mapspam.info/data/ AND https://s3.amazonaws.com/mapspam/2010/v1.1/readme_v1r1_global.txt](https://s3.amazonaws.com/mapspam/2010/v1.1/readme_v1r1_global.txt) | Take the physical production area of all crops in a given grid cell and calculate the proportion their production makes up. Then determine the group of crops (see table) that is most predominant. Modal disaggregate to target 5km grid |
| 2 | spam_propvegetables | Vegetable crops as proportion of crops grown in an area | continuous | Identify dominant type of crop group | [https://www.mapspam.info/data/ AND https://s3.amazonaws.com/mapspam/2010/v1.1/readme_v1r1_global.txt](https://s3.amazonaws.com/mapspam/2010/v1.1/readme_v1r1_global.txt) | Take the physical production area of all crops in a given grid cell and calculate the proportion their production makes up. Then determine the group of crops (see table) that is most predominant. Modal disaggregate to target 5km grid |
| 2 | spam_propothercrops | Other crops as proportion of crops grown in an area | continuous | Identify dominant type of crop group | [https://www.mapspam.info/data/ AND https://s3.amazonaws.com/mapspam/2010/v1.1/readme_v1r1_global.txt](https://s3.amazonaws.com/mapspam/2010/v1.1/readme_v1r1_global.txt) | Take the physical production area of all crops in a given grid cell and calculate the proportion their production makes up. Then determine the group of crops (see table) that is most predominant. Modal disaggregate to target 5km grid |
| 2 | fieldsize_ha | Average maximum field size in a given area | continuous | Is the field size of the largest size (as identified by dataset) | <https://onlinelibrary.wiley.com/doi/full/10.1111/gcb.14492> | All classes with combined with the area of cropland (both in ha) for a given grid cell. It was then assumed that the maximum field size is set to the target grid unless there is fewer cropland area. Afterwards aligned to 5km grid |
| 2 | ruminant_lsudens | Average density of ruminant livestock units (LSU) in a given area | continuous | Intensively managed areas having high stocking rate of ruminants | [https://www.nature.com/articles/sdata2018227 and http://www.lrrd.org/lrrd18/8/chil18117.htm](http://www.lrrd.org/lrrd18/8/chil18117.htm) | Take global layer of areal-weighted density of ruminants, apply region-specific livestock unit conversion factors, then calculate the density per area at 5km grid cell resolution |
| 2 | otherlivestock_lsudens | Average density of other livestock (pigs, chicken, ducks, horses) in livestock units in a given area | continuous | Areas with high numbers of chicken or ducks likely posses good supply chains | <https://www.nature.com/articles/sdata2018227> | Take global layer of areal-weighted density of other livestock, apply region-specific livestock unit conversion factors, then calculate the density per area at 5km grid cell resolution |
| 2 | spam_propirrigated | Fraction of land that has irrigated harvested crops (SPAM) | continuous | Delineate predominantly irrigated crop areas | <https://www.mapspam.info/data/> | Take physical areas of production of all crops per grid cell (sum). Calculate the proportion and then the most predominantly used cultivation mode (irrigated or rainfed). Resample to 5km |
| 2 | spam_proprainfed | Fraction of land that has rainfed harvested crops (SPAM) | continuous | Delineate predominantly rainfed crop areas | <https://www.mapspam.info/data/> | Take physical areas of production of all crops per grid cell (sum). Calculate the proportion and then the most predominantly used cultivation mode (irrigated or rainfed). Resample to 5km |

S2 Table. **Grouping of SPAM crops into dominant crop group categories**. Crop yield data from the SPAM database was aggregated according to specific crop types.

| cropname | crop_grouping |
| --- | --- |
| wheat | Cereals and Oilcrops |
| rice | Cereals and Oilcrops |
| maize | Cereals and Oilcrops |
| barley | Cereals and Oilcrops |
| pearl millet | Cereals and Oilcrops |
| small millet | Cereals and Oilcrops |
| sorghum | Cereals and Oilcrops |
| other cereals | Cereals and Oilcrops |
| potato | Tubers |
| sweet potato | Tubers |
| yams | Tubers |
| cassava | Tubers |
| other roots | Tubers |
| bean | LegumesPulses |
| chickpea | LegumesPulses |
| cowpea | LegumesPulses |
| pigeonpea | LegumesPulses |
| lentil | LegumesPulses |
| other pulses | LegumesPulses |
| soybean | Cereals and Oilcrops |
| groundnut | Cereals and Oilcrops |
| coconut | Perennials |
| oilpalm | Perennials |
| sunflower | Cereals and Oilcrops |
| rapeseed | Cereals and Oilcrops |
| sesameseed | Cereals and Oilcrops |
| other oil crops | Cereals and Oilcrops |
| sugarcane | Perennials |
| sugarbeet | Cereals and Oilcrops |
| cotton | Other |
| other fibre crops | Other |
| arabica coffee | Perennials |
| robusta coffee | Perennials |
| cocoa | Perennials |
| tea | Perennials |
| tobacco | Other |
| banana | Perennials |
| plantain | Perennials |
| tropical fruit | Perennials |
| temperate fruit | Perennials |
| vegetables | Vegetables |
| rest of crops | Perennials |

S3 Table. **Summary table of estimated area for each foodscape class in the map.** Estimated global area (million ha) and proportion (*100) of all land area for each foodscape class.

| ID | Label | Area (million ha) | Area (% of all land area) |
| --- | --- | --- | --- |
| 1 | Areas with little or only subsistence food production | 4542.564 | 31.385 |
| 2 | Highly urbanized land | 7.8632 | 0.054 |
| 3 | Inland water | 98.69447 | 0.682 |
| 101 | Entisols on plains with bare land, little food production and grass cover | 155.8085 | 1.077 |
| 102 | Entisols on plains with grazed bare land and grass cover | 1340.961 | 9.265 |
| 103 | Entisols on plains with bare land and scattered mixed crop production and low nutrient application rate | 492.2113 | 3.401 |
| 104 | Entisols on dry plains and large cultivated fields and livestock | 141.7719 | 0.98 |
| 105 | Entisols on dry rainfed plains with legumes and pulses production and occassionaly other crops | 11.42908 | 0.079 |
| 109 | Entisols on dry plains and bare land with mixed irrigated crop production | 5.879953 | 0.041 |
| 110 | Entisols on dry plains and bare land with irrigated vegetable production and high nutrient application rates | 21.86291 | 0.151 |
| 201 | Inceptisols on humid hilly tree-covered land with scattered crop production | 237.8299 | 1.643 |
| 202 | Inceptisols on humid mountaineous land with tree cover and scattered mixed crop production | 87.36729 | 0.604 |
| 203 | Inceptisols on humid hilly-mountains with tree cover and small farmed mixed and intensive diverse production | 247.0909 | 1.707 |
| 204 | Inceptisols on humid forested hills with intensive mixed crop production and grazing | 170.4188 | 1.177 |
| 205 | Inceptisols on humid hilly mixed tree-covered land with rainfed perennial crops and other livestock | 16.59563 | 0.115 |
| 206 | Mixed rainfed highly productive land with agroforestry and diverse crops | 32.81043 | 0.227 |
| 209 | Inceptisols on humid land with intensive mixed perrenial tree crops and non-ruminant grazing | 20.83438 | 0.144 |
| 210 | Inceptisols on humid hilly land with intensive mixed livestock and other crops grown with high nutrient application rates | 56.12817 | 0.388 |
| 301 | Mixed urban and peri-urban areas with some agriculture and livestock | 5.571561 | 0.038 |
| 304 | Peri-urban areas with marginal agriculture and livestock | 39.25472 | 0.271 |
| 306 | Peri-urban area interspersed with intensive irrigated agriculture and livestock | 38.02381 | 0.263 |
| 401 | Mollisols in mountaineous bare areas with little crop production and grazing | 24.11708 | 0.167 |
| 402 | Mollisols in mountaineous-hilly areas with low density livestock grazing and scattered crop production | 465.8359 | 3.219 |
| 403 | Mollisols in mountaineous-hilly cultivated land with grazing ruminants and rainfed mixed crops | 150.091 | 1.037 |
| 404 | Mollisols in hilly conventionally tillaged cultivated land with interspersed grazing | 184.438 | 1.274 |
| 406 | Mollisols and Inceptisols in plains with irrigated intensive crop production | 88.94226 | 0.615 |
| 407 | Mollisols in plains with intensive irrigated cereal and oil crop production and high nutrient application rates | 159.2041 | 1.1 |
| 408 | Mollisols in intensive rainfed cereal and oil crop producing land with high nutrient application rates | 39.96326 | 0.276 |
| 409 | Mollisols in plains with intensive rainfed large field with cereal and oil crop production | 538.0894 | 3.718 |
| 410 | Mollisols in plains with intensive rainfed cereal and oil crop producing land that is single cropped | 158.4266 | 1.095 |
| 501 | Vertisols in plains with grazed shrubby land and scattered mixed crop production | 140.5355 | 0.971 |
| 504 | Vertisols in plains diversely cultivated land and interspersed grazing | 59.33363 | 0.41 |
| 506 | Vertisols in plains with mixed crop and livestock production | 25.5636 | 0.177 |
| 508 | Vertisols in plains with mixed irrigated and rainfed production with mixed crop production | 90.5797 | 0.626 |
| 509 | Vertisols in plains with rainfed intensively cultivated land with mixed production and sparse grazing | 36.75008 | 0.254 |
| 510 | Vertisols in plains with larger intensively cultivated fields with reduced tillage | 52.81454 | 0.365 |
| 601 | Inceptisols on bare grassy land with scattered grazing | 140.1562 | 0.968 |
| 602 | Inceptisols on mixed forest and grassland | 745.3093 | 5.149 |
| 603 | Inceptisols in hilly grassy land with scattered grazing and marginal crop production | 51.72357 | 0.357 |
| 604 | Inceptisols in mountaineous bare land with small fields and traditional tillage | 42.93079 | 0.297 |
| 605 | Inceptisols in forested land with few scattered large farms and low crop diversity | 33.71676 | 0.233 |
| 606 | Inceptisols in hilly land with mixed production of conventional tillage and high nutrient application | 35.52718 | 0.245 |
| 607 | Inceptisols in arid hilly land with rainfed cereal and legume production and other livestock | 1.703494 | 0.012 |
| 609 | Inceptisols in hills and mountains with irrigated intensive mixed crop production | 2.890154 | 0.02 |
| 610 | Inceptisols in hilly shrubland with irrigated intensive mixed crop production and high nutrient application | 1.073555 | 0.007 |
| 701 | Alfisols in plains and grasslands with little crop production and grazing | 0.998755 | 0.007 |
| 702 | Alfisols in shrubby plains that are grazed with scattered cropland | 257.8294 | 1.781 |
| 703 | Alfisols in mixed food production landscapes with scattered grazing | 110.0886 | 0.761 |
| 704 | Alfisols in mixed diverse crop systems on small fields with some livestock and agroforestry and low nutrient application rates | 262.174 | 1.811 |
| 705 | Alfisols with mixed crop production, some ruminants, and higher nutrient application rates | 245.4311 | 1.696 |
| 706 | Alfisols with rainfed crop production on large fields with some livestock | 209.8863 | 1.45 |
| 707 | Alfisols with rainfed diverse crop production with some livestock | 113.5081 | 0.784 |
| 708 | Alfisols with irrigated intensive mixed crop production and ruminants | 54.35702 | 0.376 |
| 709 | Alfisols with mixed irrigated intensive cereal production and livestock with high nutrient application rates | 67.31076 | 0.465 |
| 710 | Alfisols with rainfed intensive cereal production and livestock with high nutrient application rates | 66.15644 | 0.457 |
| 801 | Andisols on bare land with little crop production | 0.761146 | 0.005 |
| 802 | Andisols on hilly land with little crop production | 13.90429 | 0.096 |
| 803 | Andisols on hilly and mountainous land with sparse crop production and ruminants | 3.625062 | 0.025 |
| 804 | Andisols on hilly tree and shrub land with scattered crop production | 2.702483 | 0.019 |
| 901 | Histosols and Spodosols on wet mountainous land with little crop production | 2.718121 | 0.019 |
| 902 | Spodosols on hilly tree-covered land with scattered crop production | 542.315 | 3.747 |
| 903 | Histosols and Spodosols with rainfed mixed crop production and livestock including ruminants | 1.1982 | 0.008 |
| 904 | Histosols and Spodosols in tree-covered landscapes with scattered crop production on large fields | 42.2439 | 0.292 |
| 905 | Histosols and Spodosols on mountainous land with grazing and interspersed food production | 9.907929 | 0.068 |
| 906 | Histosols and Spodosols on hilly tree-covered land grazed and cultivated with high nutrient application rate | 86.17815 | 0.595 |
| 909 | Histosols and Spodosols on intensively cultivated land with high livestock production | 3.452117 | 0.024 |
| 910 | Spodosols on intensively cultivated land high livestock production and nutrient application rate | 6.439473 | 0.044 |
| 1001 | Oxisols on humid tree-covered land with little food production | 0.721238 | 0.005 |
| 1002 | Oxisols and Ultisols on humid tree-covered land with scattered cropland and livestock | 86.26668 | 0.596 |
| 1003 | Oxisols and Ultisols on humid hilly tree-covered land with agroforestry and some livestock | 119.4143 | 0.825 |
| 1004 | Oxisols and Ultisols on humid tree-covered land with diverse small field production and agroforestry | 407.7901 | 2.817 |
| 1005 | Oxisols and Ultisols with rainfed perennial crops and agroforestry and some livestock | 99.39227 | 0.687 |
| 1006 | Oxisols and Ultisols with mixed grazing and crop production on large fields | 167.5528 | 1.158 |
| 1007 | Oxisols and Ultisols with rainfed perennial crops and agroforestry and high nutrient rates and livestock | 8.177336 | 0.056 |
| 1009 | Oxisols and Ultisols on land with humid rainfed and irrigated perennial production and other mixed crops and livestock | 19.65698 | 0.136 |
| 1010 | Oxisols and Ultisols on humid irrigated intensive perennial production and other mixed crops and livestock | 15.98151 | 0.11 |
| 1101 | Ultisols on humid tree-covered land with little crop production | 1.718149 | 0.012 |
| 1102 | Ultisols on humid tree-covered land with scattered crop production | 34.97104 | 0.242 |
| 1103 | Ultisols on humid tree-covered land with scattered crop production on large fields | 27.90733 | 0.193 |
| 1104 | Ultisols on humid tree-covered land with diverse crop production | 61.31509 | 0.424 |
| 1105 | Ultisols on hilly and mountainous tree-covered land with diverse crop production and high nutrient application rates | 99.57801 | 0.688 |
| 1106 | Ultisols with mixed crop and livestock production and high nutrient application rates | 23.53196 | 0.163 |
| 1107 | Ultisols on humid tree-covered land with diverse crop production and some livestock | 92.25116 | 0.637 |
| 1108 | Ultisols with mixed crops including perennials and livestock production | 14.583 | 0.101 |
| 1109 | Ultisols with intensively cultivated rainfed and irrigated mixed crop and livestock production | 9.947681 | 0.069 |
| 1110 | Ultisols with intensively cultivated rainfed and irrigated mixed crop and livestock production and high nutrient application rates | 38.86619 | 0.269 |

S4 Table. **Most dominant foodscape class per climatic zone**. Summary of unique foodscapes as well as the most dominant combination per Köppen geiger zone climate zone.

| **Köppen geiger zone** | **Unique terrestrial foodscapes** | **Most dominant foodscape class** |
| --- | --- | --- |
| Af | 76 | Areas with little or only subsistence food production |
| Am | 79 | Areas with little or only subsistence food production |
| Aw | 80 | Oxisols and Ultisols on humid tree-covered land with diverse small field production and agroforestry |
| BWh | 56 | Areas with little or only subsistence food production |
| BWk | 57 | Entisols on plains with grazed bare land and grass cover |
| BSh | 69 | Alfisols in shrubby plains that are grazed with scattered cropland |
| BSk | 79 | Mollisols in mountaineous-hilly areas with low density livestock grazing and scattered crop production |
| Csa | 70 | Alfisols with mixed crop production, some ruminants, and higher nutrient application rates |
| Csb | 77 | Inceptisols on humid forested hills with intensive mixed crop production and grazing |
| Csc | 16 | Inceptisols on mixed forest and grassland |
| Cwa | 74 | Inceptisols on humid hilly-mountains with tree cover and small farmed mixed and intensive diverse production |
| Cwb | 72 | Oxisols and Ultisols on humid tree-covered land with diverse small field production and agroforestry |
| Cwc | 17 | Mollisols in mountaineous-hilly cultivated land with grazing ruminants and rainfed mixed crops |
| Cfa | 78 | Mollisols in plains with intensive rainfed large field with cereal and oil crop production |
| Cfb | 76 | Inceptisols on humid forested hills with intensive mixed crop production and grazing |
| Cfc | 18 | Areas with little or only subsistence food production |
| Dsa | 39 | Mollisols in mountaineous-hilly areas with low density livestock grazing and scattered crop production |
| Dsb | 57 | Mollisols in mountaineous-hilly areas with low density livestock grazing and scattered crop production |
| Dsc | 35 | Inceptisols on mixed forest and grassland |
| Dsd | 4 | Inceptisols on mixed forest and grassland |
| Dwa | 51 | Mollisols in plains with intensive irrigated cereal and oil crop production and high nutrient application rates |
| Dwb | 59 | Areas with little or only subsistence food production |
| Dwc | 50 | Spodosols on hilly tree-covered land with scattered crop production |
| Dwd | 6 | Spodosols on hilly tree-covered land with scattered crop production |
| Dfa | 58 | Mollisols in plains with intensive rainfed large field with cereal and oil crop production |
| Dfb | 70 | Mollisols in plains with intensive rainfed large field with cereal and oil crop production |
| Dfc | 49 | Areas with little or only subsistence food production |
| Dfd | 11 | Inceptisols on mixed forest and grassland |
| ET | 51 | Areas with little or only subsistence food production |
| EF | 12 | Areas with little or only subsistence food production |

S5 Table. **Matchup table between foodscape classes and determined level of intensity.** Code corresponding to the mapped foodscape class and labels as well as corresponding intensity groupings and labels thereof. In addition total area estimates (in ha) are provided.

| foodscape | intensity | grouping_intensity | Label | sum_area_ha |
| --- | --- | --- | --- | --- |
| 1 | 101 | Areas with little or only subsistence food production | Areas with little or only subsistence food production | 4542564460 |
| 2 | 102 | Urbanized land | Highly urbanized land | 7863200.377 |
| 3 | 103 | Inland water | Inland water | 98694465.68 |
| 101 | 1 | Scattered cropland and grazing | Entisols on plains with bare land, little food production and grass cover | 155808537 |
| 102 | 1 | Scattered cropland and grazing | Entisols on plains with grazed bare land and grass cover | 1340960918 |
| 103 | 1 | Scattered cropland and grazing | Entisols on plains with bare land and scattered mixed crop production and low nutrient application rate | 492211302 |
| 104 | 2 | Mixed and diverse food cultivation | Entisols on dry plains and large cultivated fields and livestock | 141771866 |
| 105 | 2 | Mixed and diverse food cultivation | Entisols on dry rainfed plains with legumes and pulses production and occassionaly other crops | 11429079 |
| 109 | 3 | Irrigated and/or intensive food production | Entisols on dry plains and bare land with mixed irrigated crop production | 5879953 |
| 110 | 3 | Irrigated and/or intensive food production | Entisols on dry plains and bare land with irrigated vegetable production and high nutrient application rates | 21862913 |
| 201 | 1 | Scattered cropland and grazing | Inceptisols on humid hilly tree-covered land with scattered crop production | 237829934 |
| 202 | 1 | Scattered cropland and grazing | Inceptisols on humid mountaineous land with tree cover and scattered mixed crop production | 87367292 |
| 203 | 2 | Mixed and diverse food cultivation | Inceptisols on humid hilly-mountains with tree cover and small farmed mixed and intensive diverse production | 247090930 |
| 204 | 2 | Mixed and diverse food cultivation | Inceptisols on humid forested hills with intensive mixed crop production and grazing | 170418768 |
| 205 | 2 | Mixed and diverse food cultivation | Inceptisols on humid hilly mixed tree-covered land with rainfed perennial crops and other livestock | 16595632 |
| 206 | 2 | Mixed and diverse food cultivation | Mixed rainfed highly productive land with agroforestry and diverse crops | 32810433 |
| 209 | 3 | Irrigated and/or intensive food production | Inceptisols on humid land with intensive mixed perrenial tree crops and non-ruminant grazing | 20834378 |
| 210 | 3 | Irrigated and/or intensive food production | Inceptisols on humid hilly land with intensive mixed livestock and other crops grown with high nutrient application rates | 56128170 |
| 301 | 1 | Scattered cropland and grazing | Mixed urban and peri-urban areas with some agriculture and livestock | 5571561 |
| 304 | 2 | Mixed and diverse food cultivation | Peri-urban areas with marginal agriculture and livestock | 39254722 |
| 306 | 3 | Irrigated and/or intensive food production | Peri-urban area interspersed with intensive irrigated agriculture and livestock | 38023814 |
| 401 | 1 | Scattered cropland and grazing | Mollisols in mountaineous bare areas with little crop production and grazing | 24117083 |
| 402 | 1 | Scattered cropland and grazing | Mollisols in mountaineous-hilly areas with low density livestock grazing and scattered crop production | 465835868 |
| 403 | 2 | Mixed and diverse food cultivation | Mollisols in mountaineous-hilly cultivated land with grazing ruminants and rainfed mixed crops | 150091016 |
| 404 | 2 | Mixed and diverse food cultivation | Mollisols in hilly conventionally tillaged cultivated land with interspersed grazing | 184437989 |
| 406 | 3 | Irrigated and/or intensive food production | Mollisols and Inceptisols in plains with irrigated intensive crop production | 88942256 |
| 407 | 3 | Irrigated and/or intensive food production | Mollisols in plains with intensive irrigated cereal and oil crop production and high nutrient application rates | 159204108 |
| 408 | 3 | Irrigated and/or intensive food production | Mollisols in intensive rainfed cereal and oil crop producing land with high nutrient application rates | 39963265 |
| 409 | 3 | Irrigated and/or intensive food production | Mollisols in plains with intensive rainfed large field with cereal and oil crop production | 538089377 |
| 410 | 3 | Irrigated and/or intensive food production | Mollisols in plains with intensive rainfed cereal and oil crop producing land that is single cropped | 158426629 |
| 501 | 1 | Scattered cropland and grazing | Vertisols in plains with grazed shrubby land and scattered mixed crop production | 140535526 |
| 504 | 2 | Mixed and diverse food cultivation | Vertisols in plains diversely cultivated land and interspersed grazing | 59333633 |
| 506 | 2 | Mixed and diverse food cultivation | Vertisols in plains with mixed crop and livestock production | 25563603 |
| 508 | 3 | Irrigated and/or intensive food production | Vertisols in plains with mixed irrigated and rainfed production with mixed crop production | 90579698 |
| 509 | 3 | Irrigated and/or intensive food production | Vertisols in plains with rainfed intensively cultivated land with mixed production and sparse grazing | 36750076 |
| 510 | 3 | Irrigated and/or intensive food production | Vertisols in plains with larger intensively cultivated fields with reduced tillage | 52814540 |
| 601 | 1 | Scattered cropland and grazing | Inceptisols on bare grassy land with scattered grazing | 140156241 |
| 602 | 1 | Scattered cropland and grazing | Inceptisols on mixed forest and grassland | 745309343 |
| 603 | 1 | Scattered cropland and grazing | Inceptisols in hilly grassy land with scattered grazing and marginal crop production | 51723568 |
| 604 | 1 | Scattered cropland and grazing | Inceptisols in mountaineous bare land with small fields and traditional tillage | 42930789 |
| 605 | 2 | Mixed and diverse food cultivation | Inceptisols in forested land with few scattered large farms and low crop diversity | 33716760 |
| 606 | 2 | Mixed and diverse food cultivation | Inceptisols in hilly land with mixed production of conventional tillage and high nutrient application | 35527180 |
| 607 | 2 | Mixed and diverse food cultivation | Inceptisols in arid hilly land with rainfed cereal and legume production and other livestock | 1703494 |
| 609 | 3 | Irrigated and/or intensive food production | Inceptisols in hills and mountains with irrigated intensive mixed crop production | 2890154 |
| 610 | 3 | Irrigated and/or intensive food production | Inceptisols in hilly shrubland with irrigated intensive mixed crop production and high nutrient application | 1073555 |
| 701 | 1 | Scattered cropland and grazing | Alfisols in plains and grasslands with little crop production and grazing | 998755 |
| 702 | 1 | Scattered cropland and grazing | Alfisols in shrubby plains that are grazed with scattered cropland | 257829396 |
| 703 | 1 | Scattered cropland and grazing | Alfisols in mixed food production landscapes with scattered grazing | 110088633 |
| 704 | 2 | Mixed and diverse food cultivation | Alfisols in mixed diverse crop systems on small fields with some livestock and agroforestry and low nutrient application rates | 262173969 |
| 705 | 2 | Mixed and diverse food cultivation | Alfisols with mixed crop production, some ruminants, and higher nutrient application rates | 245431081 |
| 706 | 2 | Mixed and diverse food cultivation | Alfisols with rainfed crop production on large fields with some livestock | 209886301 |
| 707 | 3 | Irrigated and/or intensive food production | Alfisols with rainfed diverse crop production with some livestock | 113508056 |
| 708 | 3 | Irrigated and/or intensive food production | Alfisols with irrigated intensive mixed crop production and ruminants | 54357015 |
| 709 | 3 | Irrigated and/or intensive food production | Alfisols with mixed irrigated intensive cereal production and livestock with high nutrient application rates | 67310760 |
| 710 | 3 | Irrigated and/or intensive food production | Alfisols with rainfed intensive cereal production and livestock with high nutrient application rates | 66156442 |
| 801 | 1 | Scattered cropland and grazing | Andisols on bare land with little crop production | 761146 |
| 802 | 1 | Scattered cropland and grazing | Andisols on hilly land with little crop production | 13904290 |
| 803 | 1 | Scattered cropland and grazing | Andisols on hilly and mountainous land with sparse crop production and ruminants | 3625062 |
| 804 | 1 | Scattered cropland and grazing | Andisols on hilly tree and shrub land with scattered crop production | 2702483 |
| 901 | 1 | Scattered cropland and grazing | Histosols and Spodosols on wet mountainous land with little crop production | 2718121 |
| 902 | 1 | Scattered cropland and grazing | Spodosols on hilly tree-covered land with scattered crop production | 542314982 |
| 903 | 2 | Mixed and diverse food cultivation | Histosols and Spodosols with rainfed mixed crop production and livestock including ruminants | 1198200 |
| 904 | 2 | Mixed and diverse food cultivation | Histosols and Spodosols in tree-covered landscapes with scattered crop production on large fields | 42243903 |
| 905 | 2 | Mixed and diverse food cultivation | Histosols and Spodosols on mountainous land with grazing and interspersed food production | 9907929 |
| 906 | 2 | Mixed and diverse food cultivation | Histosols and Spodosols on hilly tree-covered land grazed and cultivated with high nutrient application rate | 86178146 |
| 909 | 3 | Irrigated and/or intensive food production | Histosols and Spodosols on intensively cultivated land with high livestock production | 3452117 |
| 910 | 3 | Irrigated and/or intensive food production | Spodosols on intensively cultivated land high livestock production and nutrient application rate | 6439473 |
| 1001 | 1 | Scattered cropland and grazing | Oxisols on humid tree-covered land with little food production | 721238 |
| 1002 | 1 | Scattered cropland and grazing | Oxisols and Ultisols on humid tree-covered land with scattered cropland and livestock | 86266682 |
| 1003 | 2 | Mixed and diverse food cultivation | Oxisols and Ultisols on humid hilly tree-covered land with agroforestry and some livestock | 119414303 |
| 1004 | 2 | Mixed and diverse food cultivation | Oxisols and Ultisols on humid tree-covered land with diverse small field production and agroforestry | 407790117 |
| 1005 | 2 | Mixed and diverse food cultivation | Oxisols and Ultisols with rainfed perennial crops and agroforestry and some livestock | 99392271 |
| 1006 | 2 | Mixed and diverse food cultivation | Oxisols and Ultisols with mixed grazing and crop production on large fields | 167552815 |
| 1007 | 2 | Mixed and diverse food cultivation | Oxisols and Ultisols with rainfed perennial crops and agroforestry and high nutrient rates and livestock | 8177336 |
| 1009 | 3 | Irrigated and/or intensive food production | Oxisols and Ultisols on land with humid rainfed and irrigated perennial production and other mixed crops and livestock | 19656980 |
| 1010 | 3 | Irrigated and/or intensive food production | Oxisols and Ultisols on humid irrigated intensive perennial production and other mixed crops and livestock | 15981513 |
| 1101 | 1 | Scattered cropland and grazing | Ultisols on humid tree-covered land with little crop production | 1718149 |
| 1102 | 1 | Scattered cropland and grazing | Ultisols on humid tree-covered land with scattered crop production | 34971036 |
| 1103 | 2 | Mixed and diverse food cultivation | Ultisols on humid tree-covered land with scattered crop production on large fields | 27907330 |
| 1104 | 2 | Mixed and diverse food cultivation | Ultisols on humid tree-covered land with diverse crop production | 61315094 |
| 1105 | 2 | Mixed and diverse food cultivation | Ultisols on hilly and mountainous tree-covered land with diverse crop production and high nutrient application rates | 99578014 |
| 1106 | 2 | Mixed and diverse food cultivation | Ultisols with mixed crop and livestock production and high nutrient application rates | 23531961 |
| 1107 | 2 | Mixed and diverse food cultivation | Ultisols on humid tree-covered land with diverse crop production and some livestock | 92251161 |
| 1108 | 3 | Irrigated and/or intensive food production | Ultisols with mixed crops including perennials and livestock production | 14583003 |
| 1109 | 3 | Irrigated and/or intensive food production | Ultisols with intensively cultivated rainfed and irrigated mixed crop and livestock production | 9947681 |
| 1110 | 3 | Irrigated and/or intensive food production | Ultisols with intensively cultivated rainfed and irrigated mixed crop and livestock production and high nutrient application rates | 38866192 |
